# Psychological and cultural correlates of illness conception and menopausal symptoms: a cross-sectional and longitudinal comparative study of Mosuo, Yi, and Han women

**DOI:** 10.3389/fpsyt.2025.1496889

**Published:** 2025-03-21

**Authors:** Limin Gao, Jinyi Wang, Ying Zhang, Xudong Zhao, Haojie Fu

**Affiliations:** ^1^ Clinical Research Center for Mental Disorders, Chinese-German Institute of Mental Health, Shanghai Pudong New Area Mental Health Center, School of Medicine, Tongji University, Shanghai, China; ^2^ Clinical Research Center for Mental Disorders, Shanghai Pudong New Area Mental Health Center, Tongji University, Shanghai, China; ^3^ Mental Health Education Center, Sichuan University, Chengdu, Sichuan, China; ^4^ Department of General Internal Medicine and Psychosomatics, Medical Hospital, University of Heidelberg, Heidelberg, Germany; ^5^ Shanghai Research Institute for Intelligent Autonomous Systems, Tongji University, Shanghai, China; ^6^ Shanghai Institute of Intelligent Science and Technology. Tongji University, Shanghai, China

**Keywords:** menopausal symptoms, health beliefs, illness conception, Mosuo, longitudinal comparison

## Abstract

**Objective:**

This study explores the differences in menopausal symptoms, illness conception, and health-seeking behaviors among Mosuo, Yi, and Han women in China, analyzes the key factors behind these differences, and conducts a longitudinal comparison.

**Methods:**

This study collected data from Mosuo, Yi, and Han women in Yongning Township, Ninglang County, Yunnan Province, through a questionnaire survey. The instruments included the Kupperman Menopause Index (KMI), Depression-Anxiety-Stress Scale (DASS), Self-Rating Scale of Illness Conception and Health Seeking Behavior (SSICHSB) and General Self-Efficacy Scale (GSES). First, descriptive statistical analysis was conducted on the demographic characteristics and various indicators of the sample. Chi-square tests and one-way ANOVA were used to examine the differences in KMI and SSICHSB among the different ethnic groups. The KMI was used to assess menopausal symptoms, and multiple linear regression analysis was employed to identify the main factors influencing menopausal symptoms. A longitudinal comparison of data from 2012 and 2020 was performed to analyze the dynamic changes in KMI and SSICHSB of Mosuo and Han women.

**Results:**

The regression analysis identified stress, anxiety, and dysmenorrhea experience as risk factors, while self-efficacy served as a protective factor influencing menopausal symptoms. Both the menopausal symptoms and the scores for concerns and fears about illness among Mosuo women significantly decreased in 2020 compared to 2012 (p = 0.040, p = 0.010).

**Conclusion:**

The results provide an important basis for the development of culturally sensitive health interventions. Future public health strategies should consider cultural, social, and physiological factors to provide more effective health support and interventions for women from different ethnic groups.

## 1 Introduction

Menopause is a natural physiological transition, but its symptoms (such as vasomotor symptoms and mood fluctuations) can significantly impact daily functioning and quality of life (1, 2). In addition to the symptoms associated with menopause, the accompanying biological, psychological, behavioral, and social changes also play a significant role in shaping a woman’s midlife and future health (3). In previous research, cultural factors related to menopausal symptoms have garnered significant attention from scholars (4). A report from the Study of Women’s Health Across the Nation (SWAN) indicates that White women report significantly more psychosomatic symptoms than women of other racial/ethnic groups, while African American women report significantly more vasomotor symptoms (4). From the perspective of the biocultural paradigm, menopausal symptoms vary across different countries. Compared to Western nations, Asian countries have a lower prevalence of vasomotor symptoms. However, with Japan’s increasing Westernization in recent years, the incidence of hot flashes has gradually risen (5). Cross-cultural comparisons reveal that women in matrilineal societies, such as the Mosuo of China, typically report milder menopause symptoms and lower rates of depression compared to those in patriarchal populations like the Han Chinese (6, 7). However, these findings highlight the unique experiences of menopause, yet the exploration of the underlying cultural and psychological factors remains limited. The limitations of current research primarily focus on symptoms, overlooking underlying cultural mechanisms such as social systems, spiritual beliefs, and health perceptions. This has sparked the interest of researchers, particularly from the perspective of psychosomatic medicine, leading to curiosity about cultural factors influencing menopausal symptoms.

The Mosuo represent the only matrilineal society in China and one of the few matriarchal societies that have endured since ancient times (8), with a population of approximately 53,000, the Mosuo people are primarily concentrated around Lugu Lake, a scenic area located in the canyon region on the eastern edge of the Tibetan Plateau and the western part of Sichuan Province, near the border between Yunnan and Sichuan provinces (9). The Mosuo people are officially classified as a branch of the Naxi ethnic group in China’s official ethnic classification system (6). At the heart of Mosuo society is a matrilineal system in which inheritance and lineage pass through women. Households are structured as extended families led by elder women who hold significant authority. A defining feature of the Mosuo is their unique “walking marriage” system, where relationships are free from economic ties or moral obligations (10). This tradition shapes a distinctive family structure that sets them apart from other societies. Additionally, the matrilineal family structure of the Mosuo people emphasizes females without diminishing the importance of males, and the Mosuo people are generally religious. These cultural characteristics may significantly influence the experiences of Mosuo women during menopause. The constraints of gender norms and the impact on women’s autonomy can increase the risk of chronic diseases (11). In contrast, the Yi people follow a strictly patrilineal system, with lineage and property inheritance entirely based on the male line. As neighbors, the Mosuo and Yi share a comparable overall economic, social, and political environment despite their contrasting kinship structures (9). This cultural divergence offers a unique opportunity to explore how societal structures and health beliefs mediate menopausal symptomatology, making it an ideal context for a cross-cultural comparison study on menopause symptoms.

There are various theoretical frameworks that underpin assumptions about the influence of sociocultural factors on menopausal symptoms. First, sociocultural theory posits that cultural background and social environment profoundly influence an individual’s psychology and behavior (12). The unique culture of the Mosuo may shape their perceptions and approaches to illness. Second, psychosocial development theory emphasizes the impact of family structure and social support on an individual’s mental health (13). The Mosuo people maintain a unique lifestyle and belief system, which deeply influences their health concepts and help-seeking behaviors (14). The matrilineal family structure and religious beliefs of the Mosuo may provide a special support system for women, thus affecting their menopausal experiences. Finally, the biopsychosocial model integrates biological, psychological, and social factors, suggesting that these factors collectively influence health status (15). The impact of hormonal changes during menopause on depressive mood (16) is intricately interconnected with past experiences of depression, stress, and significant life events, forming a complex interplay of influences. The hormonal changes that begin during the menopausal transition affect multiple biological systems, leading to psychological changes (16). Menopause often coincides with significant life stressors, changes in health status, and role transitions, all of which heighten vulnerability to depression (17). Individuals with anxiety or a negative perception of menopause may develop maladaptive cognitive appraisals of vasomotor symptoms, such as fearing that others will notice their hot flashes or believing that their symptoms will never subside. These negative perceptions can exacerbate psychological distress and amplify the impact of these symptoms on emotional well-being and daily life (18). Self-efficacy can mitigate the impact of menopausal symptoms on life satisfaction (19). Therefore, we selected these culturally relevant factors as the focus of our study.

Whether individuals perceive menopause as a disease and their attitudes toward it are equally important. Concerns about heightened risks of anxiety and depression may influence both the expectations and experiences of menopause (18). Illness perception refers to the process by which patients use their prior knowledge and experiences with illness to analyze and interpret current symptoms or conditions (20). This theoretical foundation is rooted in Leventhal’s Common-Sense Model of Self-Regulation, developed in the 1980s. Leventhal’s model suggests that people adapt to threats through effective coping mechanisms and accurate perceptions of their environment (21, 22). The attitudes and beliefs that emerge during this process are often referred to as illness conception (20). Although menopause is not considered a disease, different illness perceptions can lead to varying impacts on psychosomatic health issues. Help-seeking behavior, also known as illness behavior, is the act of seeking medical assistance with the goal of preventing disease, detecting early symptoms, or pursuing treatment (23). While illness represents an objective reality, illness conception is the subjective understanding of this reality, and help-seeking behavior is the outward manifestation of this understanding. To investigate the influence of cultural differences on menopausal symptoms, as well as variations in illness conception and help-seeking behaviors across different contexts, this study compares three groups of women from distinct cultural backgrounds: Mosuo, Yi, and Han. We hypothesize that the differences in menopausal symptoms and help-seeking behavior among Mosuo women are primarily influenced by their unique cultural factors.

Menopausal experiences are profoundly shaped by cultural context, yet most research has focused on Western or urban populations. This study examines the Mosuo, a matrilineal ethnic group in Southwest China, whose cultural frameworks offer a unique perspective on menopause as a non-pathological transition. This study aims to systematically explore the differences in menopausal symptoms, health concepts, and help-seeking behaviors among Mosuo, Yi, and Han women, and the key factors underlying these differences. To achieve this goal, the research is divided into three main sections:

First, we will compare the differences in menopausal symptoms and illness conception and help-seeking behaviors among Mosuo, Yi, and Han women. Through this part of the study, we aim to reveal the physiological and psychological characteristics of women during menopause under different cultural backgrounds.

Second, we will analyze the key factors influencing the severity of menopausal symptoms. By identifying the influence of variables such as cultural background, age, and education level on menopausal symptoms, we can determine which factors play a crucial role in menopausal symptoms.

Finally, we will conduct a longitudinal comparison between Mosuo and Han women, analyzing the dynamic changes in menopausal symptoms, illness conception and help-seeking behaviors. Through longitudinal comparison, we hope to understand the long-term impact of social and cultural changes on women’s health behaviors.

The comprehensive analysis of this study will not only contribute to the scientific understanding of menopausal symptoms but also provide practical guidance for improving the health status of women from different cultural backgrounds. By deeply understanding the interaction of cultural, social, and physiological factors, we can more effectively support women through menopause and improve their quality of life.

## 2 Participants and methods

### 2.1 Participants

The research area for this study is Yongning Township, Ninglang County, Yunnan Province, a region where Mosuo, Yi, and Han ethnic groups traditionally reside together (10). Yongning Township is renowned for its diverse cultural background and unique ethnic traditions. According to data from the local police station, there are a total of 2,038 women aged 40 to 60 in this area, including 1,071 Mosuo women, 316 Yi women, and 651 Han women (24). As the main settlement of the Mosuo people, Yongning Township provides an ideal setting for studying the impact of cultural factors on menopausal symptoms and help-seeking behaviors.

The inclusion criteria for participating in this study were Mosuo, Yi, and Han women aged 40 to 60 who volunteered to participate. The exclusion criteria were women who had undergone unnatural menopause, those using medications that affect endocrine function, those with a history of severe physical or mental illness, and those unable or unwilling to participate in the study. During the research process, a fixed researcher used standardized scales to evaluate all participants, assisted by a young Mosuo woman fluent in both the Mosuo language and Mandarin, with at least a middle school education, who served as a translator. In the end, a total of 210 valid questionnaires were collected, including 81 from Mosuo women, 71 from Yi women, and 58 from Han women.

### 2.2 Methods

#### 2.2.1 General information questionnaire

In this study, we utilized a self-designed questionnaire to systematically gather demographic data and cultural characteristics of the participants. This questionnaire included basic demographic information such as age, height, and smoking and alcohol consumption habits. Additionally, it addressed cultural and socioeconomic variables including ethnicity, religious beliefs, and education level. We also explored participants’ roles in household financial management, family decision-making styles, and overall family status. Furthermore, we assessed participants’ awareness and understanding of menopause, as well as their experiences with menstrual pain. Detailed information regarding the General Information Questionnaire can be found in **Supplementary Material 1**.

#### 2.2.2 Kupperman menopause index

The tool used to evaluate menopausal symptoms was the Kupperman Menopause Index (KMI), which consists of 13 items (25). KMI provides a comprehensive assessment of the severity of menopausal syndrome by integrating physical, psychological, and autonomic symptoms. Each symptom is assigned to a different weight, but to preserve the independent clinical significance of each symptom, they are not combined. The modified Chinese version of the KMI has been demonstrated to be effective in identifying and assessing discomfort during the climacteric period (26). In this study, considering local cultural taboos, the item “sexual intercourse difficulty” was removed. Each item’s score was determined by multiplying the symptom severity by the symptom index. Symptom severity was graded on a scale from 0 (none) to 3 (severe), and symptoms with a severity score of ≥1 were considered present. The symptom index was categorized based on weights of 4, 2, and 1. Hot flashes and sweating were assigned the weight of 4. Paresthesia, insomnia, nervousness, and urinary symptoms were weighted as 2. Other symptoms were given a weight of 1. The total score was calculated by summing up all weighted symptom scores, with higher total scores indicating more severe menopausal symptoms.

#### 2.2.3 Self-rating scale of illness conception and health seeking behavior

This study adopted the Self-Rating Scale of Illness Conception and Health Seeking Behavior (SSICHB), developed by Su Ping et al. (20). The questionnaire consists of 16 items divided into three subscales: “Development of Illness Conception and Influencing Factors in Childhood” (6 items), where higher scores indicate a stronger influence of family members on illness beliefs and a greater tendency to focus on and fear diseases; “Conception of Disease and Health in Adulthood” (7 items), where higher scores suggest increased concern and fear about illness, along with a more pessimistic attitude toward disease; and “Help-Seeking Methods and Behaviors” (3 items), where higher scores reflect a proactive approach to illness management, emphasizing the role of personal effort in recovery. It uses a five-point Likert scale: 1 = fully agree; 2 = mostly agree; 3 = somewhat agree; 4 = mostly disagree; 5 = fully disagree. The total score for each factor is the sum of the scores for the included items (with 2 reverse-scored items among the 16 items). The subdimension “Perception and Attitude towards Illness and Health in Adulthood” includes items such as “Even if I feel that there is something wrong with my health, I am reluctant to seek medical attention.”

#### 2.2.4 Depression, anxiety, and stress

The Chinese version of the Simplified Depression-Anxiety-Stress Self-Rating Scale (DASS-21) was utilized in this study (27). The DASS-21 is a shortened version of the original DASS (28), consisting of 21 items divided into three subscales: Depression, Anxiety, and Stress, with each subscale containing seven items. Responses are rated on a four-point Likert scale: 0 = not at all applicable, 1 = somewhat applicable, 2 = mostly applicable, 3 = completely applicable. Higher total scores indicate greater levels of negative emotional experiences.

#### 2.2.5 General self-efficacy scale

This study utilized the General Self-Efficacy Scale (GSES) to measure participants’ self-efficacy. Originally version developed by Schwarzer (29), the scale has been adapted into a Chinese version and consists of 10 items (30). It employs a 4-point Likert scale, with total scores ranging from 10 to 40, where higher scores reflect greater perceived self-efficacy.

### 2.3 Statistical methods

Data analysis was conducted using SPSS 27.0 statistical software. First, descriptive statistics were performed on the demographic characteristics and various indicators of the sample, including frequency, percentage, mean, and standard deviation calculations. To examine the differences in menopausal symptoms and help-seeking behaviors among women from different ethnic groups, Chi-square tests and one-way analysis of variance (ANOVA) were used. The severity of menopausal symptoms was assessed using the Kupperman Menopause Index, and the total score of the Kupperman Index was used to measure symptom severity. Then, in a multiple linear regression analysis, the KMI was treated as the dependent variable, while cultural and psychological factors, including the subdimensions of SSICHB, DASS-21, and GSES, were included as independent variables. Height and age were treated as control variables. All statistical analyses were two-tailed tests, with a significance level set at p < 0.05.

## 3 Results

### 3.1 Common method bias validation

The results of Harman’s single-factor test showed that 24 factors with eigenvalues greater than 1 were extracted from the included features, collectively explaining 73.054% of the total variance. The variance explained by the first factor was 16.105%, which did not exceed 40%. Therefore, there is no significant common method bias in this study (31).

### 3.2 Description and comparison of demographic characteristics of Mosuo, Yi, and Han participants

The average age of the Mosuo, Yi, and Han women (49.543 ± 5.266, 49.686 ± 7.056, 49.483 ± 4.612, respectively) showed no statistically significant difference (F = 0.022, p = 0.979). And there was a significant difference in height (F = 14.211, p < 0.001), the Mosuo women were found to be taller than the Han and Yi women. Almost all surveyed Mosuo women have religious beliefs, such as Daba and Tibetan Buddhism. Yi women primarily follow local traditional beliefs, with Bimo and Suni being important figures in religious and cultural activities, while the proportion of religious adherence among Han women is very low.

According to the statistical analysis results in **Table 1**, there were significant differences in several demographic characteristics between the different ethnic groups. Firstly, there was no significant difference in menopause awareness rates among the three ethnic groups. After conducting an ANOVA on education level, the results indicated significant differences among the ethnic groups (F = 6.059, p = 0.003). *Post-hoc* comparisons revealed that the education level of the Han ethnic group was significantly higher than that of the Yi group (p = 0.029). Similarly, the education level of the Mosuo group was significantly higher than that of the Yi group (p < 0.001). Smoking status also showed significant differences (χ² = 32.614, p < 0.001), with Yi women having a significantly higher smoking rate (31.0%) compared to Mosuo (6.2%) and Han (0.0%) women. And the difference in alcohol consumption habits among the three groups was not statistically significant (χ² = 2.919, p = 0.232). There were significant differences in dysmenorrhea experiences among the different ethnic groups (χ² = 9.196, p = 0.010), with the highest proportion reported by Han (62.1%) women. There were no statistically significant differences in personal income control and family income support among the different ethnic groups (χ² = 3.282, p = 0.194; χ² = 1.625, p = 0.444). These results provide foundational data for further research on the impact of different cultural backgrounds on menopausal symptoms.

**Table 1:** Description and comparison of demographic characteristics of mosuo, Yi, and Han participants.

Variable	Mosuo (N=81)	Yi (N=71)	Han (N=58)	χ^2^/F	p
Religious Belief					
Tibetan Buddhism	54 (66.7%)	0 (0.0%)	0 (0.0%)		
Daba Religion	15 (18.5%)	1 (1.4%)	1 (1.7%)		
Bimo, Suni	0 (0.0%)	62 (87.3%)	0 (0.0%)		
Others	1 (1.2%)	0 (0.0%)	4 (6.9%)		
None	18 (22.2%)	11 (15.5%)	56 (96.6%)		
Heard of Menopause				4.559	0.102
Yes	29 (35.8%)	37 (52.1%)	27 (46.6%)		
No	52 (64.2%)	33 (46.5%)	31 (53.4%)		
Education Level				6.059	.003
Illiterate	58 (71.6%)	66 (93.0%)	43 (74.1%)		
Primary School or Below	15 (18.5%)	4 (5.6%)	12 (20.7%)		
Middle School	6 (7.4%)	1 (1.4%)	3 (5.2%)		
High School	2 (2.5%)	0 (0.0%)	0 (0.0%)		
Smoking Status				32.614	<0.001
Yes	5 (6.2%)^a^	22 (31.0%)^b^	0 (0.0%)^a^		
No	76 (93.8%)^a^	49 (69.0%)^b^	58 (100.0%)^a^		
Alcohol Consumption				2.919	0.232
Yes	7 (8.6%)	5 (7.0%)	1 (1.7%)		
No	74 (91.4%)	66 (93.0%)	57 (98.3%)		
Dysmenorrhea				9.196	0.010
Yes	32 (39.5%)^a^	27 (38.0%)^a^	36 (62.1%)^b^		
No	49 (60.5%)^a^	44 (62.0%)^a^	22 (37.9%)^b^		
Controls Personal Income				3.282	0.194
Yes	74 (91.4%)	60 (84.5%)	47 (81.0%)		
No	7 (8.6%)	11 (15.5%)	11 (19.0%)		
Controls Family Income					
Yes	60 (74.1%)	53 (74.6%)	38 (65.5%)	1.625	0.444
No	21 (25.9%)	18 (25.4%)	20 (34.5%)		

The percentages in parentheses represent the proportion of each category within the respective cultural group. Different superscripts (a, b) indicate statistically significant differences between groups (p < 0.05). Groups sharing the same letter do not show significant differences. Statistical comparison for Religious Belief was not performed due to excessive zero responses.

### 3.3 Comparative analysis of KMI and SSICHSB among Mosuo, Yi, and Han women

Based on the detection rates of various symptoms from the modified KMI, the top three symptoms reported by Mosuo women were paresthesia (72.8%), arthralgia/myalgia (71.6%), and headache (55.6%). For Yi women, the most common symptoms were arthralgia/myalgia (77.5%), paresthesia (71.8%), and headache (66.2%), while Han women reported paresthesia (87.9%), arthralgia, myalgia (75.9%), and headache (70.7%) as the most frequent symptoms. There were significant differences in menopausal symptoms among women of different ethnic groups. Specifically, there were significant differences among Mosuo, Yi, and Han women in the prevalence of insomnia, vertigo, fatigue, and urinary tract infection. Han women had the highest rates of insomnia (67.2%) and vertigo (69.0%), while Mosuo women had the lowest prevalence of these symptoms (35.8%, 44.4%). Yi women had the highest rates of fatigue (69.0%) and urinary tract infection (38.0%), with Mosuo women again showing the lowest prevalence (14.8%).

As shown in **Table 2**, in terms of the modified KMI total score (excluding the item on sexual intercourse difficulty), Mosuo women scored lower than Yi and Han women (10.580 ± 8.329, 13.873 ± 8.657, 16.224 ± 9.306, F = 7.355, p < 0.021, p < 0.001), with no significant difference between Yi and Han women (p = 0.129). Overall, the differences in menopausal symptoms and experiences among women from different ethnic backgrounds highlight the significant role that ethnicity plays, providing a basis for offering more culturally tailored health management and support to women.

**Table 2:** Comparative analysis of menopausal symptoms among Mosuo, Yi, and Han women.

Symptom	Overall (N=210)	Mosuo (N=81)	Yi (N=71)	Han (N=58)	χ^2^	p
Hot flushes and sweatin	103 (49.0%)	35 (43.2%)	38 (53.5%)	30 (51.7%)	1.839	0.399
Paresthesia	161 (76.7%)	59 (72.8%)	51 (71.8%)	51 (87.9%)	5.705	0.058
Insomnia	104 (49.5%)	29 (35.8%)^a^	36 (50.7%)^a,b^	39 (67.2%)^b^	13.424	0.001
Nervousness	117 (55.7%)	40 (49.4%)	39 (54.9%)	38 (65.5%)	3.593	0.166
Melancholia	87 (41.4%)	26 (32.1%)	32 (45.1%)	29 (50.0%)	5.050	0.080
Vertigo	121 (57.6%)	36 (44.4%)^a^	45 (63.4%)^b^	40 (69.0%)^b^	9.780	0.008
Fatigue	119 (56.7%)	32 (39.5%)^a^	49 (69.0%)^b^	38 (65.5%)^b^	15.972	<0.001
Arthralgia/Myalgia	157 (74.8%)	58 (71.6%)	55 (77.5%)	44 (75.9%)	0.740	0.691
Headache	133 (63.3%)	45 (55.6%)	47 (66.2%)	41 (70.7%)	3.712	0.156
Heart palpitation	97 (46.2%)	30 (37.0%)	34 (47.9%)	33 (56.9%)	5.487	0.064
Formication	43 (20.5%)	17 (21.0%)	9 (12.7%)	17 (29.3%)	5.446	0.066
Urinary tract infection	56 (26.7%)	12 (14.8%)^a^	27 (38.0%)^b^	17 (29.3%)^b^	10.712	0.005

All values in this table represent detection rates (%) for each symptom within the respective cultural group. Different superscripts (a, b) indicate statistically significant differences between groups (p < 0.05). Groups sharing the same letter do not show significant differences.

As shown in **Table 3**, based on the data from the SSICHSB scale, there were significant differences in illness conception and help-seeking behavior across different stages among Mosuo, Yi, and Han women. The overall score did not show a significant difference among the three ethnic groups (p = 0.259). However, analysis of specific dimensions revealed significant differences in the DIIC among the three groups (p = 0.001), with Yi women scoring the highest, significantly higher than Han and Mosuo women. There were also significant differences in HSMB among the three groups (p = 0.049), with Han women scoring the highest, significantly higher than Yi and Mosuo women.

**Table 3:** Comparative analysis of illness conception and help-seeking behavior among Mosuo, Yi, and Han women.

Variable	Mosuo	Yi	Han	F	p	LSD
SSICHSB	42.173 ± 8.040	43.571 ± 8.261	44.310 ± 6.924	1.359	0.259	
DIIC	16.617 ± 5.004	15.457 ± 3.973	16.845 ± 4.196	1.888	0.154	
CDHA	15.556 ± 4.117	18.000 ± 3.757	16.345 ± 4.331	6.949	<0.001	Yi>Han^*^ Yi>Mosuo^***^
HSMB	10.000 ± 3.114	10.114 ± 2.800	11.121 ± 2.340	3.051	0.049	Han> Yi^*^ Han>Mosuo^*^

*Significant at the 0.05 level. ***Significant at the 0.001 level. SICHSB refers to the Self-Rating Scale of Illness Conception and Health-Seeking Behavior; DIIC stands for the Development of illness conception and influencing factors in childhood; CDHA represents the Conception of disease and health in adulthood; and HSMB denotes Help-seeking Methods and Behaviors.

### 3.4 Regression analysis

To further explore the key factors influencing Kupperman scores, a linear regression analysis was conducted on the entire sample of women. In the regression analysis of the entire sample, we included multiple potential influencing factors such as age, education level, religious belief, lifestyle factors (e.g., smoking, drinking), psychosocial factors (e.g., DASS, GSES), and cultural background. The analysis results showed that stress, the presence of dysmenorrhea, anxiety, religious belief, and self-efficacy had significant impacts on the Kupperman score in the overall sample, providing a basis for further subgroup analysis.

Through regression analysis, we can gain a clearer understanding of the factors influencing menopausal symptoms. These results also provide scientific evidence for formulating targeted health intervention strategies.

The regression model was established by including all samples and using the stepwise method to select relevant key factors (R² = 0.568, adjusted R² = 0.552, R² change = 0.486).

As shown in **Table 4**, after controlling for age and height, five key predictors of KMI were identified for Mosuo, Yi, and Han women: stress, dysmenorrhea, anxiety, religious belief, and self-efficacy.

**Table 4:** Linear regression analysis results of factors influencing KMI in the entire sample.

	Model (Selected Factors)	B	SE	β	t	P	VIF
**Control Variables**	(Constant)	13.065	12.970		1.007	0.315	
	Age	.117	.079	.074	1.483	0.140	1.118
	Height	-.171	.074	-.116	-2.296^*^	0.023	1.118
**Independent Variables**	DASS_Stress	.819	.186	.347	4.417^***^	<0.001	2.691
	Dysmenorrhea	3.281	.943	.179	3.481^***^	<0.001	1.156
	DASS_Anxiety	.925	.236	.296	3.925^***^	<0.001	2.480
	Religious Belief	2.395	.939	.129	2.551^*^	0.012	1.107
	Self-Efficacy	-.126	.057	-.109	-2.205*	0.029	1.072

*Significant at the 0.05 level. ***Significant at the 0.001 level.

DASS_Anxiety refers to the Anxiety subscale of the Depression Anxiety Stress Scale (DASS), DASS_stress refers to the Stress subscale of the DASS. KMI refers to the Kupperman Menopause Index.

Among the independent variables, stress levels (DASS_Stress) and the experience of dysmenorrhea both had significant effects on the Kupperman score—higher stress levels and the presence of dysmenorrhea were associated with more severe menopausal symptoms. Additionally, anxiety levels (DASS_Anxiety) also had a significant effect on the Kupperman score. The presence of religious belief and self-efficacy also significantly influenced menopausal symptoms. Specifically, women without religious beliefs and those with lower self-efficacy experienced more severe menopausal symptoms. These findings suggest that psychological factors and physiological experiences play important roles in the severity of menopausal symptoms.

### 3.5 Longitudinal comparison

To explore the changes in menopausal symptoms, health perceptions, and help-seeking behaviors among Mosuo women, this study compared data collected in 2012 and 2020. By longitudinally comparing the data from these two time points, we analyzed the dynamic changes in these aspects among Mosuo women over time.

As shown in **Table 5**, by comparing the data collected in 2012 and 2020, it was found that Mosuo women had significantly lower menopausal symptoms (Kupperman score) in 2020 compared to 2012 (p = 0.040), indicating a reduction in their menopausal symptoms. Additionally, Mosuo women had significantly lower scores in the dimension of CDHA in 2020 compared to 2012 (p = 0.010), suggesting a decrease in their concern and worry about illness. There were no significant changes in the dimensions of DIIC and HSMB.

**Table 5:** Comparative analysis of data collected in 2012 and 2020.

Variable	Mosuo					Han				
	2012 (N=51)	2020 (N=81)	t	P	d	2012 (N=47)	2020 (N=58)	t	p	d
KMI	13.3 ± 7.0	10.580 ± 8.329	2.072^*^	0.040	0.346	16.6 ± 8.5	16.224 ± 9.306	0.214	0.831	0.042
DIIC	16.8 ± 3.7	16.617 ± 5.004	0.241	0.810	0.040	16.4 ± 5.0	16.845 ± 4.196	-0.496	0.621	-0.097
CDHA	17.6 ± 4.8	15.556 ± 4.117	2.603^*^	0.010	0.465	16.6 ± 3.8	16.345 ± 4.331	0.317	0.752	0.062
HSMB	10.9 ± 2.4	10.000 ± 3.114	1.866	0.064	0.290	12.1 ± 2.1	11.121 ± 2.340	2.231^*^	0.028	0.438

*Significant at the 0.05 level. KMI refers to the Kupperman Menopause Index; SICHSB refers to the Self-Rating Scale of Illness Conception and Health-Seeking Behavior; DIIC stands for the Development of illness conception and influencing factors in childhood; CDHA represents the Conception of disease and health in adulthood; and HSMB denotes Help-seeking Methods and Behaviors.

For Han women, there were no significant changes in KMI, CDHA and DIIC. However, their scores in the dimension of HSMB were significantly lower in 2020 than in 2012 (p = 0.028).

## 4 Discussion

This study analyzed the menopausal symptoms, illness perceptions, and help-seeking behaviors of Mosuo, Yi, and Han women, revealing differences in health behaviors and perceptions during menopause among women from different cultural backgrounds.

### 4.1 Ethnic differences in demographic and menopausal symptoms

The descriptive analysis revealed significant differences across various factors among Mosuo, Yi, and Han women. Notably, Han women had significantly lower rates of smoking and drinking compared to the other two groups, with smoking rates particularly high among Yi women. This finding aligns with previous research (32), which indicated that participants from ethnic minorities and those with lower education levels were more likely to smoke. Similarly, in this study, Yi women had the highest illiteracy rate, surpassing both Han and Mosuo women, highlighting significant educational disparities between the ethnic groups.

The significant differences in menopausal symptoms among Mosuo, Yi, and Han women indicate that ethnic factors play an important role in the prevalence of these symptoms. Mosuo women had the lowest scores in menopausal symptoms, indicating milder symptoms. Similar studies have suggested that the matrilineal social structure and “walking marriage” system of the Mosuo may play a positive role in reducing psychological stress among women (33). Han women had the highest rates of insomnia and vertigo, which could be attributed to the fact that many of them migrated from various parts of the country. Despite living there for some time, they may not have fully adapted to the local environment, climate, or atmospheric pressure (34). This ongoing process of cultural and environmental adaptation may lead to additional psychological and physiological stress (35), contributing to the higher rates of insomnia and vertigo. Yi women exhibited the highest prevalence of fatigue and urinary tract infection, in contrast, Mosuo women had the lowest rates of several menopausal symptoms, such as insomnia, vertigo, fatigue, and urinary tract infections. This could be linked to the unique cultural and social structure of the Mosuo community, where women may receive greater family support (7) and experience less stress (24), thus reducing the severity of menopausal symptoms.

### 4.2 Psychological and cultural factors influencing menopausal symptoms

The regression analysis further identified key factors influencing menopausal symptoms. Higher levels of stress and anxiety were associated with more severe menopausal symptoms (p < 0.001), demonstrating that psychological factors play an important role in symptom severity. This is consistent with previous research that found a close relationship between psychological stress, anxiety levels, and the severity of menopausal symptoms (36, 37). However, depression scores were not included, as there is currently no direct evidence linking depression to menopausal symptoms (18). Significant increases in depression during menopause are primarily observed in women with a history of depressive episodes (38). Stress and anxiety are significant risk factors for menopausal symptoms. Women experiencing anxiety symptoms face an increased risk of developing moderate to severe vasomotor symptoms during the menopausal transition (39). These findings support the hypothesis that psychosocial factors influence menopausal symptoms (18). Additionally, the presence of dysmenorrhea was identified as a risk factor for menopausal symptoms. Experiencing pain and discomfort may increase the risk of developing psychosomatic symptoms. Additionally, the presence or absence of religious beliefs is significantly associated with menopausal symptoms. However, as shown in **Table 1**, the differences in religious beliefs closely align with ethnic differences. Due to the small sample size, whether these variations are related to religious beliefs requires further investigation in future research. These findings highlight the importance of physiological and psychological factors in managing menopausal health, suggesting that multiple factors should be considered when developing interventions. The results indicate that self-efficacy is a protective factor against menopausal symptoms. Previous studies have found that self-efficacy moderates the relationship between symptoms and menopausal life satisfaction (19), highlighting that self-efficacy is an important psychological factor that plays a crucial role in maintaining women’s physical and mental health.

### 4.3 A cross-sectional and temporal changes in illness conception and health-seeking behavior

In the comparison of SSICHSB, Yi women scored significantly higher than both Han and Mosuo women in their conception of disease and health in adulthood, suggests that Yi women exhibit a higher level of concern and fear regarding illness compared to Mosuo and Han women. The lack of difference between Mosuo and Han women is consistent with previous findings (14), while the higher scores among Yi women support earlier research on the health-related quality of life in the Yi population (40). The high prevalence of infections among Yi women may be related to their more negative illness perceptions. Health beliefs shaped by ethnicity and culture can greatly influence related health behaviors (41).

In terms of help-seeking methods and behaviors, Han women, having received higher levels of modern education and possessing greater knowledge of modern medicine, tend to have a more optimistic and proactive attitude towards illness. They tend to believe that recovery from illness is largely dependent on their own efforts. In contrast, Mosuo and Yi women were less likely than Han women to believe that recovery from illness is significantly related to their personal efforts, which may be linked to their ethnic religious beliefs. The Mosuo people, who practice Daba or Tibetan Buddhism, often seek herbal remedies for minor illnesses, while major or unexplained ailments are treated with a combination of Western medicine and religious rituals (42). The Yi people in the Xiao-liangshan region of southwest China have a unique practice of combining ritual healing and traditional medicine for patient care, often avoiding hospitals and other modern medical facilities (43).

Comparing data from 2012 and 2020, it was found that Mosuo women experienced significantly reduced menopausal symptoms in 2020 (p = 0.040), likely due to changes in their social and cultural environment. Over the years, Mosuo women have undergone natural adaptation and physiological adjustment, gradually becoming more accustomed to the changes associated with menopause, which has naturally led to a reduction in symptoms. A decrease in the use of their traditional language (44), along with improvements in the built environment and the growth of tourism (45), has significantly altered their lifestyle. With the development of modern medicine, Mosuo women in 2012 may have confused some disease symptoms with menopausal symptoms in the survey, but now they are better able to distinguish symptom attribution and receive appropriate treatment for underlying conditions, further reducing the reported incidence of menopausal symptoms. Compared to eight years ago, Mosuo women have developed a more positive outlook on illness, reflecting the profound impact of social and cultural changes on women’s health behaviors. This also highlights the integration of traditional culture with modern health concepts during the process of modernization (46, 47). Han women, while showing a significant decrease in help-seeking methods and behaviors in 2020 compared to 2012 (p = 0.028), still maintained higher scores than Mosuo women, demonstrating a relatively proactive approach. With the advancement of basic healthcare, even in impoverished areas, effective treatments for common diseases are now available (48).

### 4.4 Key findings and public health implications

These findings reveal changes in menopausal symptoms and health behaviors among women from different cultural backgrounds, providing important references for further research. The first step is to identify modifiable risk factors (18), such as the introduction of CBT therapy and the adoption of its treatment principles, as well as implementing stress management and anxiety clarification strategies (49). By providing better medical knowledge and healthcare services to women, particularly in medically underserved minority communities, their menstrual experience can be improved and discomfort reduced (50), Mitigation of risk factors. Additionally, improving women’s education levels and encouraging regular physical exercise can enhance their self-efficacy in leveraging protective factors, empowering them to better manage their menstrual health and overall well-being (18, 51). Future research should further explore the impact of cultural and social changes on women’s health to provide more targeted health support and interventions for women from different cultural backgrounds. These interventions may include culturally sensitive health education, psychological support, and personalized medical services for different ethnic groups. By comprehensively considering cultural, psychological, and social factors, we can more effectively support women through menopause and improve their quality of life.

## 5 Conclusion

Mosuo women in 2020 exhibited a significant reduction in the severity of menopausal symptoms compared to 2012, along with a decrease in concerns and fears about illness. Through multiple linear regression analysis, psychological stress, anxiety, dysmenorrhea experience, and religious belief were identified as important factors influencing menopausal symptoms. The study results provide important evidence for developing health interventions tailored to different cultural backgrounds.

## 6 Study limitations and future research directions

This study has several limitations. First, the relatively small sample size may limit the generalizability of the results. Second, data collection relied on self-reports, which may introduce subjective bias. Additionally, the study was limited to Yongning Township in Ninglang County, Yunnan Province, and may not fully represent other regions. Finally, while this study explored the impact of cultural background on menopausal symptoms and help-seeking behaviors, it did not delve into the specific cultural mechanisms.

Future research should increase the sample size to include women from more regions and different cultural backgrounds to improve the generalizability and reliability of the results. Additionally, a combination of quantitative and qualitative research methods should be used to explore how cultural, social, and psychological factors interact to influence women’s health behaviors and perceptions. Further longitudinal studies will help understand the long-term impact of cultural changes on women’s health behaviors, providing a basis for developing more effective public health strategies. Through multidisciplinary collaboration, integrating perspectives from psychology, sociology, and medicine, we can gain a more comprehensive understanding of women’s menopausal health issues and develop more effective interventions.

## Data Availability

The raw data supporting the conclusions of this article will be made available by the authors, without undue reservation.
