# Supplementary material for: Psychological and cultural correlates of illness conception and menopausal symptoms: a cross-sectional and longitudinal comparative study of Mosuo, Yi, and Han women
Source: Front Psychiatry. 2025 Mar 21;16:1496889. doi: 10.3389/fpsyt.2025.1496889 (PMC11968659; doi:10.3389/fpsyt.2025.1496889)
Supplement: Supplementary file 1 [file DataSheet1.pdf]

General Information Questionnaire [English version]

1. Date of Birth: ___ Year ___ Month

2. Height: ___ cm

3. Ethnicity:_____

4. Mosuo Group: [Single choice] *

○ Yes
○ No

5. Religious Belief:

○ Tibetan Buddhism
○ Daba
○ Bimo, Suni
○ Others
○ None

6. Have you heard of Menopause?

○ Yes
○ No

7. Education Level: [Single choice] *

○ Illiterate
○ Primary School
○ Middle School
○ High School
○ University and Above

8. Do you smoke? [Single choice] *

○ Yes
○ No

9. Do you drink alcohol? [Single choice] *

○ Yes
○ No

10. Do you experience menstrual pain? [Single choice] *

○ Yes
○ No

11. Do you control your personal income? [Single choice] *

○ Yes
○ No

12. Do you control your family income? [Single choice] *

○ Yes
○ No
